# “Student‐led workshop strengthens perceived discussion skills and community in an interdisciplinary graduate program”

**DOI:** 10.1096/fba.2021-00165

**Published:** 2022-10-28

**Authors:** Catharine Shipps, Kyra L. Thrush, Clorice R. Reinhardt, Sara A. Siwiecki, Jennifer L. Claydon, Dorottya B. Noble, Corey S. O'Hern

**Affiliations:** ^1^ Integrated Graduate Program in Physical and Engineering Biology Yale University New Haven Connecticut USA; ^2^ Department of Molecular Biophysics and Biochemistry Yale University New Haven Connecticut USA; ^3^ Graduate Program in Computational Biology and Bioinformatics Yale University New Haven Connecticut USA; ^4^ Poorvu Center for Teaching and Learning Yale University New Haven Connecticut USA; ^5^ Combined Graduate Program in Biological and Biomedical Sciences Yale University New Haven Connecticut USA; ^6^ Program in Physics, Engineering, and Biology Yale University New Haven Connecticut USA; ^7^ Department of Mechanical Engineering & Materials Science Yale University New Haven Connecticut USA; ^8^ Department of Physics Yale University New Haven Connecticut USA

**Keywords:** curriculum improvement, discussion workshop, evaluation and assessment, graduate training program, interdisciplinary training, science communication skills

## Abstract

The Integrated Graduate Program in Physical and Engineering Biology (IGPPEB) at Yale University brings together Ph.D. students from the physical, engineering, and biological sciences. The main goals of this program are for students to become comfortable working in an interdisciplinary and collaborative research environment and adept at communicating with scientists and nonscientists. To fill a student‐identified learning gap in engaging in inclusive discussions, IGPPEB students developed a communication workshop to improve skills in visual engagement, citing specific content, constructive conversation entrances, and encouragement of peers. Based on short‐ and long‐term assessment of the workshop, 100% of students reported that it should be offered to future cohorts and 63% of students perceived it to be personally helpful. Additionally, 92% of participants reported using one or more of the core skills beyond the course, with skills in “Encouraging peers” and “Constructive conversation entrances” rated the highest in perceived improvement. Based on the highest average rating of 76 ± 24 (on a scale of 0–100), students agreed that the workshop made them feel more welcome in the IGPPEB community. With a rating of 68 ± 13, they also agreed that the workshop had a positive impact on their graduate school experience. Participants provided suggestions for future improvements, such as increasing student involvement in leading discussions of course material. This study demonstrates that a student‐led workshop can improve perceived discussion skills and build community across an interdisciplinary program in the sciences.

## 1 INTRODUCTION

Discussion courses have been used successfully in a variety of settings, such as in ethics courses^1^, ^2^, ^3^ and when bringing together multiple disciplines.^4^, ^5^ The main goals of discussion courses align well with the goals of graduate education, where students take responsibility for their own learning and synthesize material beyond simple recall.^6^, ^7^ However, like learning to be an independent researcher, effective discussion‐based learning demands allocated practice time to communicate expectations for successful discussions.^6^

The discussion‐based course “Methods and Logic in Interdisciplinary Research” (see Appendix S1 for the course syllabus) serves as part of the required cross‐departmental course curriculum in Yale University's Integrated Graduate Program in Physical and Engineering Biology (IGPPEB).^8^ The course aims to train scientists to excel in interdisciplinary team‐based research to tackle complex problems in biology. This discussion‐based course was carefully constructed to address both program goals and student needs. Students are assigned research articles at the intersection of physics, engineering, and biology by faculty members who possess complementary research expertise to provoke academic discussions.^6^, ^9^ For example, a physicist and a biologist may discuss the application of the same technique in two different contexts and a theoretician and experimentalist may discuss two different approaches to the same biological problem.^10^, ^11^, ^12^, ^13^, ^14^, ^15^, ^16^, ^17^, ^18^ Peer learning, via student‐only discussions, is an important part of the course.^19^ Students meet as peers for 2 h 1 day to explore the details of the papers. A second day is used to discuss the papers with a pair of IGPPEB faculty members. It has been shown that combining both peer discussion and instructor explanation can lead to improvements in active learning compared to only using instructor explanations.^20^, ^21^ Despite these efforts, several years after the course's inception, two students approached IGPPEB leadership to report a substantial gap in learning. The students reported inefficient group discussions due to uneven participation, interruptions or dismissive comments, and fragmented conversations about key points, particularly in student‐only sessions.

To improve group discussions and overall learning, these students proposed the development of a student‐led discussion workshop, offered at the beginning of the course, so that all students can build their scientific discussion skills. The workshop builds on the Harkness method ideology and focuses on four communication skills: visual engagement, citing the text, constructive conversation, and encouragement of peers.^6^, ^22^ These skills aim to help students learn to “listen for discussion” and address a common question as a whole, rather than debate, recite facts, or discuss fragmented topics.^23^

This article is designed to communicate the development of the workshop, and assess the workshop's impact on perceived discussion skills and community building. We describe the details of how the workshop was integrated into the IGPPEB curriculum, and then analyze student feedback from workshop evaluations, including strengths and suggestions for improvement. Finally, we discuss ways in which this workshop can contribute to community building within the IGPPEB and why surveying both student needs and engagement plays an important role in the success of graduate training programs.

## 2 MATERIALS AND METHODS

### 2.1 Development of the discussion workshop

The IGPPEB leadership worked with the two Ph.D. students during Fall 2018 to develop a student‐led discussion workshop based on the Harkness method. The development of the workshop was an iterative process between the IGPPEB leadership and Ph.D. students, which encouraged student‐designed content and allowed close mentorship by IGPPEB leadership. At an initial meeting, the two student leaders explained their ideas for a workshop based on best practices for scientific discussions.^24^ The IGPPEB leadership asked the students to put together a proposal that included a syllabus, learning objectives, and lesson plans. The proposal also outlined how the workshop will benefit students and included a plan to sustain the workshop after the current student leaders graduate. Subsequent discussions between the students, IGPPEB leadership, and the Assistant Director of Training Program Assessment in Yale's Poorvu Center for Teaching and Learning, established assessment materials for participants before and after they take the course. IGPPEB leadership attended the first iteration of the workshop and provided feedback to the workshop leaders immediately after the sessions. IGPPEB leadership also continued to provide mentorship to the workshop leaders to respond to student feedback, develop plans for offering the workshop in subsequent years, and shorten it for online learning during COVID‐19 (Spring 2021).

### 2.2 Goals and format of the discussion workshop

**FIGURE 1:**
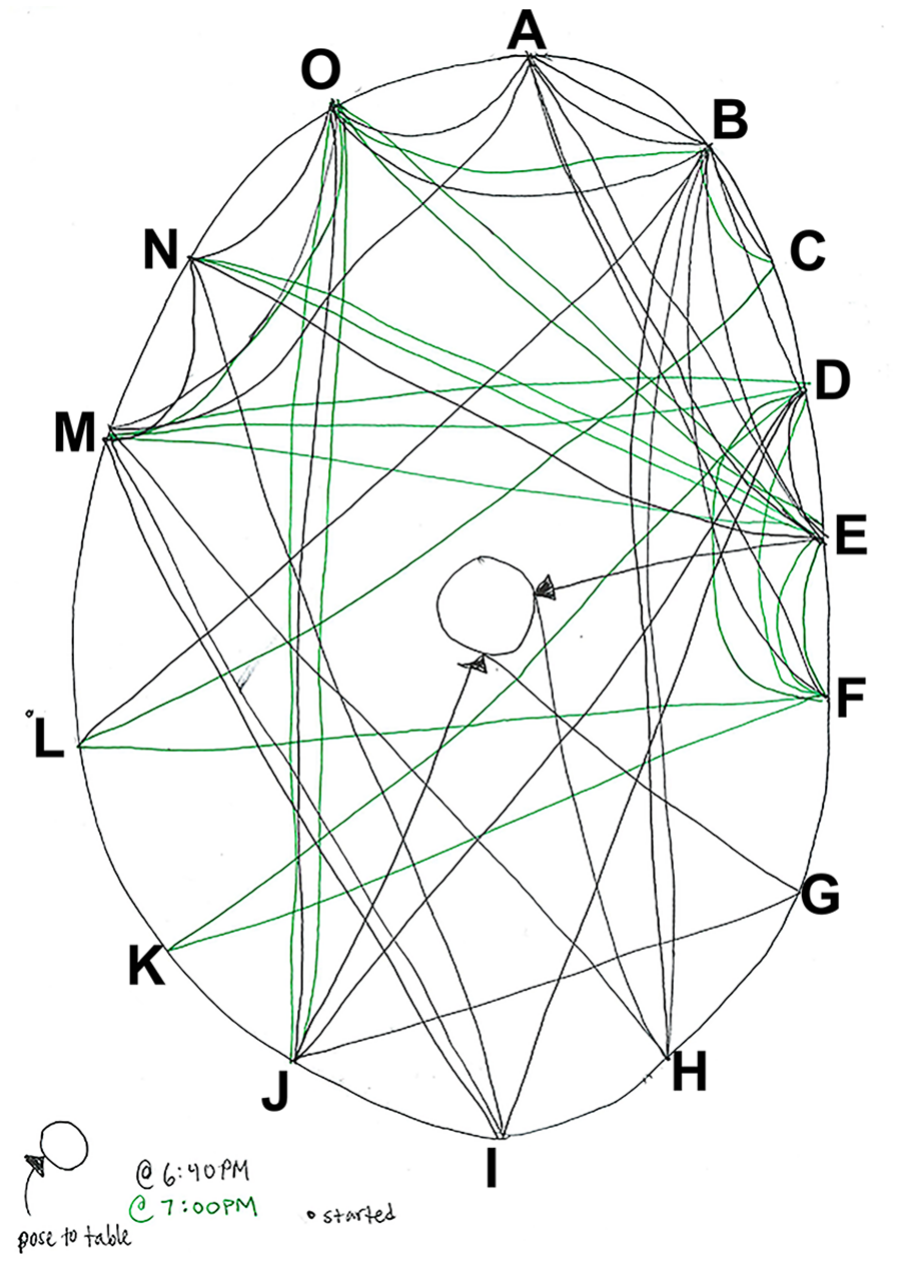
An example Harkness Discussion Diagram used during each session of the discussion workshop to provide feedback on student participation. To maintain student anonymity, students are labeled A–O. The dot next to student L indicates that this student started the discussion. Lines with an arrowhead indicate a question that was posed to the group (indicated by the central circle). Each line indicates communication between two students, denoted by the letters at the ends of the line, or a student posing a question to the group. Black lines indicate discussion contributions at the beginning of the session, and green lines indicate contributions that were made 20 min later.

The goals of the workshop include improving the discussion skills of the course participants, developing close relationships among IGPPEB students during the course, and providing an opportunity for interested IGPPEB students to improve their teaching skills by leading the workshop as discussion leaders. Via a series of community building and conversation‐intensive activities and discussions of science‐related articles, the four‐session workshop focuses on: (1) Visual engagement, which includes addressing peers by name, making eye contact while speaking, and avoiding speaking exclusively to discussion leaders; (2) Citing specific content of the articles that were read, which ensures that everyone knows the section of text, figure, or table that is referenced in the discussion; (3) Constructive conversation entrances, which helps students build on other students' ideas; and (4) Encouragement of peers, which creates balanced discussions among all course participants. The 90‐min sessions are split into an activity and a discussion portion. (See Appendix S1 for the material covered in each of the four sessions of the discussion workshop.) The student leaders moderate the activity section, but are silent during the discussion portion, which helps modulate the authoritative voice in the classroom and places that role on the students who discuss the articles. Student moderators often sit a short distance away from the workshop participants to accentuate this role.^23^ The student discussion leaders chart conversations to track student engagement using Harkness Discussion Diagrams. See Figure 1 for an example discussion diagram from the workshop. The discussion diagrams are shared with students at the end of the session and posted online with the course materials to enable them to set goals for their future scientific discussions.^6^ Discussion leaders then suggest ways to improve the discussion dynamics based on the discussion chart, for example, by pointing out if a small group dominated the discussion or if someone was silent, and identifying ways to allow space for everyone to contribute. Discussion diagrams were not analyzed as part of the study.

Another important goal of this workshop series is to provide leadership opportunities for students within the IGPPEB and help them take ownership of their training. IGPPEB students lead the workshop and refine its content for a two‐year term. During the first year, while the student leaders are new, they work with an experienced leader from the prior year. In year two, they mentor another new leader. This format introduces a peer mentoring element and allows for knowledge transfer from year to year.

### 2.3 Assessment methods

Students who took the course during the spring semesters of 2019 and 2020—the first 2 years in which the discussion workshop was integrated with the course—filled out three anonymous online surveys developed by the graduate student discussion leaders in consultation with IGPPEB leadership and Yale's Poorvu Center for Teaching and Learning. The surveys were aligned with the goals of the workshop series to allow feedback on these goals. The first survey was taken prior to the start of the discussion workshop (pre‐course survey), the second one at the end of the class (post‐course survey), and the last one in the summer of 2021 (long‐term survey). (See Appendix S2 for copies of the surveys.) The pre‐course survey focused on assessing how much prior experience students have with discussion‐based courses and whether they believe that such a workshop would help them. The post‐course survey focused on assessing whether participants believe that the workshop was helpful and in what ways, and identifying methods for improving the workshop. In both the pre‐ and post‐course surveys, questions directly examined the related variables of comfort with speaking in class, asking for help, initiating new discussion topics, and making eye‐contact during discussions. The long‐term survey focused on learning how students use the skills introduced during the workshop, 1–2 years after the course, and sought additional suggestions for improvement. Question formats included multiple choice, slider rating scales, and open‐ended short answers. Slider rating scales ranged from 0–100, where 0 indicates strongly disagree and 100 indicates strongly agree. All participants consented to their data being aggregated and summarized for IGPPEB leadership to improve to the cours and to using the data for publication purposes. This study was approved by Yale's Institutional Review Board #2000032507.

### 2.4 Data analysis and statistics

A total of 28 students (12 in 2019, 16 in 2020) were enrolled in the course and the discussion workshop.

The pre‐ and post‐course surveys were sent through anonymous links with Google Forms software. Responses for all surveys were collected anonymously to glean the most honest, accurate self‐reporting for the variables examined.

The long‐term survey was designed for students 1–2 years after taking the discussion course. Responses were collected via an anonymous online link through Qualtrics software. Due to the anonymous nature of the surveys, we were unable to directly compare whether initial demographic variables of undergraduate experiences factored into changes in ratings of comfort in the post‐course or long‐term survey.

Quantitative questions were aggregated and analyzed in Excel, SPSS, or Matlab software. We examined, using one‐way analysis‐of‐variance (ANOVAs), whether the level of prior experience with discussion‐based courses, or whether perceptions of the workshop influenced any of the pre‐course comfort levels (speaking in class, group projects, making eye contact, initiating a new discussion topic, and asking for help). Tukey's HSD test and p‐values for multiple comparisons were used to determine significance.

Qualitative responses were thematically coded in NVIVO software. Grounded theory was utilized to identify themes from the open‐ended questions.

In the Results section, unless otherwise noted, much of the data is reported as the mean ± standard deviation.

## 3 RESULTS

### 3.1 Pre‐course survey

The overall response rate for the pre‐surveys over the two course years was 100%.

#### 3.1.1 Comfort levels

**FIGURE 2:**
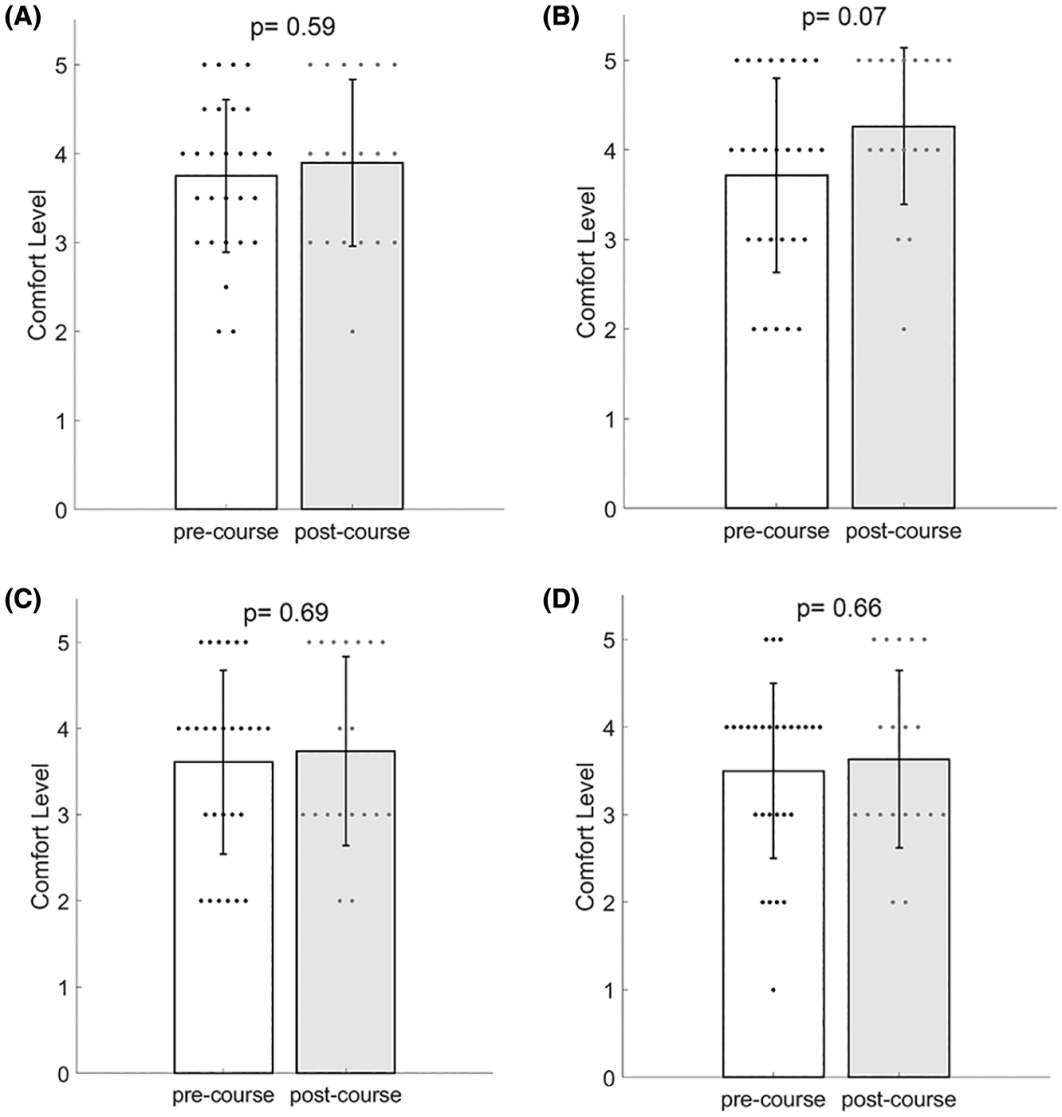
The comfort levels of students concerning (A) participation in class discussions, (B) maintaining eye contact, (C) initiating discussion on a new topic, and (D) asking for help as reported in the pre‐course (white) and post‐course (gray) surveys. The mean and standard deviation are shown, pre‐ and post‐course, with individual data points represented as dots. The p‐values from t‐tests of each corresponding pre‐ and post‐course comfort levels are also reported.

Students reported that their pre‐participation comfort with respect to speaking in class was 3.75 ± 0.86, eye contact was 3.71 ± 1.08, starting a new topic was 3.61 ± 1.07, and asking for help was 3.50 ± 1.00. (See Figure 2.) Some students already had experience with discussion‐centered courses, with only 21% reporting that ‘most of their classes’ taken at their undergraduate institution were discussion‐centered courses. Students had taken 2.21 ± 1.99 discussion‐centered courses on average with a range from 0 to 8 per student.

#### 3.1.2 Prior experience and perception of workshop

There was a statistically significant difference in comfort level when initiating a new discussion topic (*F* (2,25) = 6.071, *p* = 0.007, ANOVA, where *F*(2,25) is the F‐value, reporting the numerator and denominator degrees of freedom, and *p* is the p‐value) between students who had no prior experience with discussion‐based courses (average comfort level rating of 2.5), and those who had either taken 1–2 courses (average comfort level rating of 3.75) or 3 or more courses (average comfort level rating of 4.1). According to this finding, the more experience a participant had coming into the discussion‐based workshop series, the more comfortable they were with initiating new discussion topics in class.

Participants (*n* = 27) also responded to the open‐ended question “what experiences in academic discussion have you had before and how comfortable are you in such a setting?”. Of the respondents, 56% (*n* = 15) reported having prior experiences in graduate level discussion courses, 30% (*n* = 8) reported having prior experiences in undergraduate level courses, and 26% (*n* = 7) had prior experiences during journal clubs within their labs. Furthermore, 37% (*n* = 10) of participants stated that they felt comfortable in discussion settings, while 11% (*n* = 3) stated that they were not comfortable, and cited a desire for this discussion course to help them to build confidence. See selected quote from the pre‐course survey below:

“During undergrad, I was able to take a graduate level course where we read and discussed papers related to foundations of biology. I was very intimidated…I am hoping to build some confidence with this class.”

Participants also rated their initial perception of the discussion workshop, with 46% (*n* = 13) stating that it would be helpful, 43% (*n* = 12) stating they were not sure if it would be helpful, 3% (*n* = 1) stating that it would not be helpful, and 7% (*n* = 2) stating that it would be helpful, but not for them personally (ANOVA, *p* > 0.05), with no statistical significance between pre‐ and post‐course ratings. (See Table 1.)

**TABLE 1:** Pre‐ and post‐course survey perceptions of the helpfulness of the workshop

Perception	% respondents in pre‐survey	% respondents in post‐survey
Helpful	46% (*n* = 13)	63% (*n* = 12)
Good idea but not helpful for them	3% (*n* = 1)	31% (*n* = 6)
Not helpful	7% (*n* = 2)	0%
Not sure whether helpful or not	43% (*n* = 12)	5% (*n* = 1)

### 3.2 Post‐course survey

The response rate for the post survey over the two course years was 68% (*n* = 19). 100% of the respondents thought that the discussion workshop should be offered in subsequent years.

#### 3.2.1 Comfort levels

Participants' post‐participation comfort with speaking in class was 3.89 ± 0.94, with eye contact was 4.26 ± 0.87, with starting a new topic was 3.74 ± 1.10; and comfort with asking for help was 3.63 ± 1.01. (See Figure 2.) Although the biggest difference for pre‐ and post‐course comfort levels was seen for eye contact, none of the pre‐ and post‐course means were statistically different from each other. (See Figure 2.) Given the anonymous survey collection method, we could not examine students' pair‐wise differences within subjects.

#### 3.2.2 Perception of workshop

**FIGURE 3:**
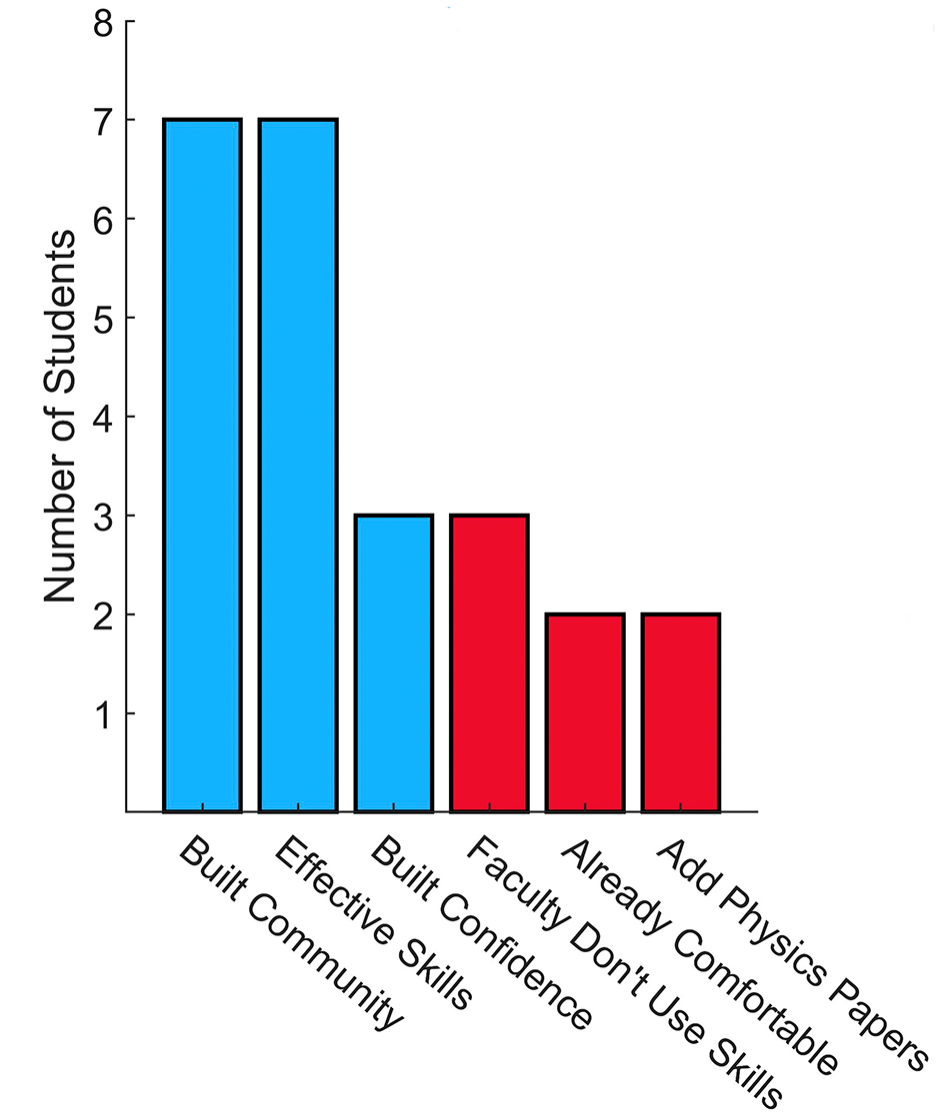
The results of the post‐course survey describing the way in which the workshop was helpful (blue) and not helpful (red).

Overall, 63% of respondents (*n* = 12) reported that they thought the workshop was helpful, 31% (*n* = 6) thought it was a good idea, but it did not help them personally, and 5% (*n* = 1) remained unsure about whether it was helpful to them. (See Table 1.) Participants responded to “why was the workshop helpful or not helpful” with 74% of students (*n* = 14) stating that the workshop was helpful in various ways. (See Figure 3.) The top themes (each accounting for 37% of participants, *n* = 7) for rating the discussion course as helpful were (1) making students feel more comfortable and welcome during the discussion, in large part because they became acquainted with each other during the workshop, and (2) because participants believe that the workshop made them reflect on how to have an effective discussion. A non‐native English speaker additionally commented that they thought they gained valuable speaking experience and skills. See sample quotes from the post‐course survey below:

“[The course] got everyone thinking about how to have an effective discussion.”

“It helped me be more confident in myself in general and in asking questions about interdisciplinary topics which helped in other discussion classes and in lab settings.”

Two of the main reasons why some students thought that the workshop was a good idea but did not help them personally, was because (1) they did not think that the skills were practiced enough during the faculty‐led course discussions (16%), and (2) they already felt comfortable with scientific discussions (11%), for example, one participant responded “I'd already felt pretty comfortable speaking up in a discussion, but getting to know my peers was certainly useful.”

#### 3.2.3 Suggestions for future improvements

Of the students, 61% (*n* = 17) suggested future improvements for the discussion workshop. (See Table 2.) The responses included mostly suggestions on format, such as using the research articles from course discussions with faculty rather than new research articles or tertiary literature during practice discussions (*n* = 6); lessening the amount of time spent on games (*n* = 2), but also implementing new games (i.e., to encourage improved listening skills so that discussion points brought up by someone can be better addressed, *n* = 4); and incorporating skills that focus on asking for help in understanding unfamiliar material and how to be an effective discussion leader (*n* = 5).

**TABLE 2:** Suggested future improvements to the workshop based on post‐course and long‐term surveys

Change	% of respondents desiring the change	Survey
Use of research articles in discussion workshop	35% (*n* = 6)	Post course
Changes to games	35% (*n* = 6)	Post‐course
More emphasis on asking for help and leading discussions	29% (*n* = 4)	Post‐course
More diverse research papers	36% (*n* = 5)	Long‐term
Faculty training in skills	29% (*n* = 4)	Long‐term
More practice with skills during the course	21% (*n* = 4)	Long‐term
Administrative changes to course	21% (*n* = 4)	Long‐term

Additional suggestions included informing course professors about the skills covered during the discussion workshop, with one student suggesting faculty also receive training on these skills. Receiving some background knowledge prior to faculty‐led discussions would help students better understand the material and participate in the discussions, since the topics covered are extremely diverse by virtue of the course objectives (*n* = 3). Furthermore, many students suggested more student involvement in the course, not only in the workshop. For example, students, along with professors, could moderate the discussion and suggest scientific articles for review (*n* = 2). Current literature has found similar suggestions for enhanced student input into discussion materials in a course for medical students.^4^

### 3.3 Longitudinal survey

**FIGURE 4:**
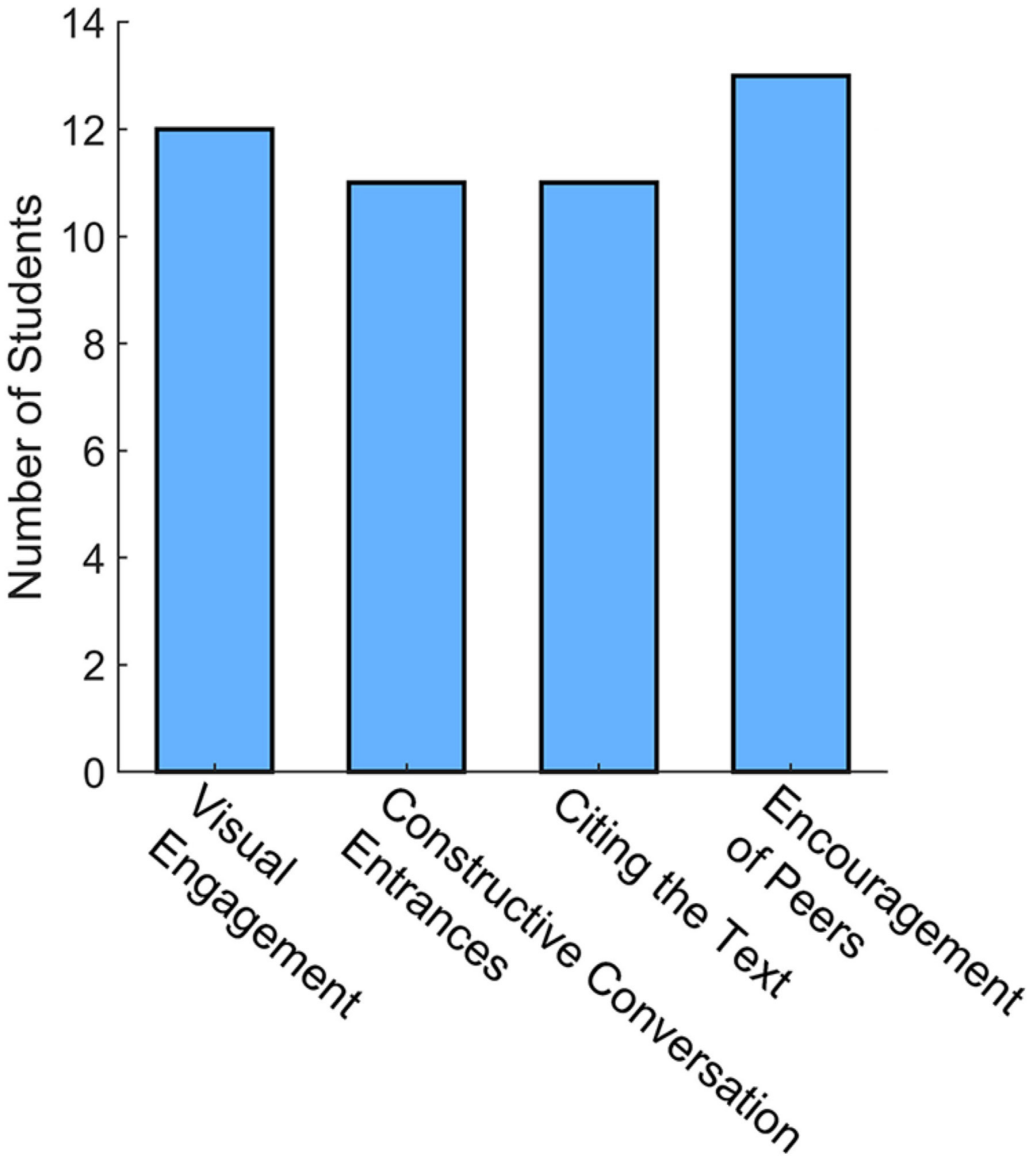
Number of students who actively used discussion skills that were covered during the discussion workshop as determined by the long‐term survey.

The response rate for the long‐term survey of the 28 students was 86% (*n* = 24). Students were asked whether they actively use the four major skills that they practiced during the discussion workshop, and 92% of students (*n* = 22) reported using one or more skills covered during the discussion workshop beyond the course. (See Figure 4.) The average slider ratings were 62 ± 11, 71 ± 16, 65 ± 11, and 72 ± 28 for visual engagement, constructive conversation entrances, citing the text, and encouraging peers, respectively, indicating overall positive experiences. (See Table 3.) Students reported using these skills in a variety of settings, such as during lab meetings, presentations, journal club discussions of research, and in discussions with undergraduates as a part of their teaching assistant duties. (See Table 3.) When asked where they use these skills, one student responded, “Everywhere, I realized how important active listening was in ALL conversations.”

**TABLE 3:** Long‐term survey results on improvements in discussion skills as a result of the workshop, and current implementation of these discussion skills

Skill	Average improvement (slider 0–100) ± SD	% responses at or above 50	Context in which the skill is implemented	Representative quote
Visual engagement	62 ± 11	91%	Lab and research meetings; Teaching Assistant duties; presentations	“When making figures, discussing concepts and drawing diagrams in lab meetings or in presentations.”
Constructive conversation entrances	71 ± 16	100%	Lab meetings; casual one‐on‐one meetings in lab; Teaching Assistant duties; discussions with students	“Everywhere; I realized how important this was to active listening in ALL conversations.”
Citing the text	65 ± 11	100%	Presentations; lab Slack channel; lab and subgroup meetings; any type of writing; journal clubs	“I use this skill in presentations but also when asking questions following a talk or in a group discussion of a paper.”
Encouraging peers	72 ± 28	92%	Talking to lab mates; journal clubs; feedback session for talks; lab meetings	“I use this skill often in all journal club experiences, both in class and in lab environments as well as in feedback sessions of talks.”

The overall degree of impact of the workshop was rated at 68 ± 13, with only positive experiences reported. Participants, on average, also reported being better prepared for scientific discussions (65 ± 25) and having more constructive conversations (63 ± 25). Furthermore, they believed that participating in this workshop helped them feel more welcome in the IGPPEB community (76 ± 24). (See Table 4 and Supporting Information for the long‐term survey instrument).

**TABLE 4:** Long‐term survey results on the impact of the discussion workshop

How the workshop has helped the participant	Average (slider 0–100) ± SD	% responses at or above 50
Overall degree of impact	68 ± 13	100%
Feel more prepared for scientific discussion	65 ± 25	84%
Have more constructive conversations	63 ± 25	84%
Feel more welcomed in the IGPPEB community	76 ± 24	89%

Of the participants, 58% (*n* = 14) responded to the open ended question “what would you change about the workshop?” Four main themes emerged from the responses. (See Table 1.) These included 36% of participants (*n* = 5) stating that they would like to see more diverse papers with emphasis on physics topics, 29% of participants (*n* = 4) stating that they believe the course discussions would be improved if faculty were also trained in these skills, 21% of participants (*n* = 3) stating that they would like more practice with these skills during the course itself, and 21% of participants (*n* = 3) had administrative comments, such as holding this course at the end of second year, adjusting the timing for when the course is offered, and including a moderator of discussions during the course.

## 4 DISCUSSION

This article describes the perceptions of student participants before and after a series of four student‐led workshop sessions aimed at developing discussion skills and community in an interdisciplinary graduate program. Developing discussion and communication is often neglected in graduate education, which has prompted many recent initiatives,^25^, ^26^ including “bottom‐up” efforts, as described in this study.

### 4.1 Impact of the workshop on precieved discussion skills

After having taken the workshop and associated course, based on the post‐course survey, all participants believed that the workshop series should be offered for future cohorts. While there were no significant differences in self‐evaluations of discussion comfort levels in the pre‐ and post‐course surveys (Figure 2.), 63% (*n* = 12) rated the workshop as helpful in the post‐course surveys. (See Table 1.) The majority of the participants reported some improvement in the four core discussion skills from the workshop (Table 3) and 92% (*n* = 22) reported using one or more skills covered during the discussion workshop beyond the course. (See Figure 4.) Respondents also cited a variety of contexts across graduate student training where they implement the discussion skills, such as in other courses, laboratory research, during presentations, and while serving as teaching assistants. (See Table 3.) It is also important to note that in the pre‐course survey, 37% (*n* = 10) of the participants stated that they felt comfortable in discussion settings, which may explain, in part, why the average pre‐ and post‐course skill ratings did not increase significantly. “Encouraging peers” and “Constructive conversation entrances” were the skills rated the highest in perceived improvement (Table 3.), which are key to improving scientific discussions and academic culture. After 1–2 years, all participants rated the overall impact of the workshops positively, with most reporting that they use the discussion skills (Figure 4) and, feel more prepared for scientific discussion, have more constructive conversations, and feel more welcomed in the graduate program community. (See Table 4.) Community building and learning effective communication skills were also cited as the most helpful aspects of the workshop. (See Figure 3.)

In a similar vein, a student led 10‐week science communication workshop reported positive impacts based on participant self reporting and external evaluations, with self‐confidence only improving minimally compared to other areas.^26^ Another study of early career researchers analyzed feelings of self‐efficacy in both oral and written communication and found that vicarious experiences were undervalued ways to develop self‐efficacy in communication skills.^27^ In this framework, vicarious experiences allow the scientist to learn by observing, or watching the communication skill being modeled or used in front of them. It is possible that building confidence in using discussion skills gained during the discussion workshop takes more time beyond that of the course, including learning from and evaluating other scientists. This may explain, in‐part, why we did not see a significant difference in self‐evaluations of discussion comfort levels in the post‐course survey (Figure 2) despite students recognizing the overall utility of the workshop (i.e. 100% of participants believed it should be offered again) as well as the discussion skills and impacts of the workshop one to two years after receiving the workshop and course training. (See Tables 3 and 4.)

Some students, however, did report “building confidence” as one of the ways the workshop was helpful. (See Figure 3.) Having confidence in leading scientific discussions and being familiar with peers can boost students' confidence to participate in additional professional development activities that lead to transferrable skills for a variety of STEM careers. Examples of such professional development activities, which the IGPPEB offers, are organizing monthly discussion groups, career events and socials, preparing for program‐specific conferences and symposia, mentoring undergraduate students conducting research in IGPPEB laboratories as part of a Yale NSF Research Experiences for Undergraduates Site managed by IGPPEB leadership, and creating IGPPEB specific science outreach events. Providing continued opportunities for students to interact and work together is an important component to building a tight‐knit program cohort.

We also found that the level of prior experience with discussion‐based courses influenced only how comfortable participants were with initiating new discussion topics during the workshop. We believe that understanding the prior experience levels of future cohorts can help to guide the student discussion leaders in their content planning, perhaps providing additional resources to those students who have no prior experience with discussion‐based courses.

### 4.2 Benefits of the workshop for graduate training programs

While the discussion workshop serves to provide Ph.D. students with important discussion skills, it also provides other benefits. In addition to “effective skills”, “community building” was also cited as one of the top ways in which the workshop was helpful. (See Figure 3.) In fact, the highest rated perceived impact, with an average of 76 ± 24 (out of 100) (Table 4), was feeling more welcome in the IGPPEB by becoming more acquainted with peers. While it is also possible to meet other students during program socials and in various non‐academic contexts, the advantage of the workshop setting is that students become acquainted with everyone, both those who are socially outgoing and those who are reserved. This social aspect of the workshop and course is especially important for the IGPPEB, which includes students from 12 departments, encompassing a broad range of research expertise and backgrounds.

Additionally, for the discussion leaders, the workshop provides an opportunity to develop their leadership and teaching skills. Participants are able to work with more junior students in the training program and contribute to IGPPEB curriculum development and assessment. Empowering students to be involved in training program activities and building a strong cohort can lead to high student retention rates, successful program recruiting, and attracting program alumni to participate in career events.

### 4.3 Student self‐awareness

It is important to note that the discussion workshop was created because IGPPEB Ph.D. students identified a gap in their skillset. The students brought the issue to the attention of IGPPEB leadership and developed the discussion workshop to improve student discussion skills. In addition, the delivery of the workshop through group activities emphasized self‐evaluation and peer recognition, which may also explain why “Encouraging peers” and “Constructive conversation entrances” were the skills rated the highest in preceived improvement. (See Table 3.) This process demonstrates that not only are the IGPPEB students assessing their training needs, through an inclusive lens, more than before, but they are also more comfortable expressing those needs. Students feeling supported in the classroom environment has been shown to improve community, motivation, and engagement in various programs.^28^, ^29^, ^30^ Thus, we suggest that it is beneficial to foster a climate where students can freely express their academic needs to both improve graduate training programs and retain students in these programs.

### 4.4 Importance of frequent student assessment in building a more inclusive learning environment

Offering this discussion workshop illustrates the importance of surveying students regularly so that program leadership can learn about suggested improvements, as well as make decisions about curriculum refinement and successfully monitor whether those changes are beneficial. Using student data as formative feedback to create improvements within the program is a positive expression of the value that leadership places on student input.^31^ All Yale courses ask students to fill out course evaluations, which evaluate the instructor of record, provide an overall assessment of the course, how the course was organized, the workload and intellectual challenge of the course, and comments on strengths, weaknesses, and any improvements to the course. However, the standard course evaluations are not course specific and therefore do not ask students to evaluate the discussion workshop or the specific skills gained by the workshop. Standard course evaluations also do not provide longer term feedback 1–2 years after the course. By using the additional surveys, (see Appendix S2), course‐specific information can be obtained as shown in Table 2. For example, without the survey results in this study, we would not have known that many students believed that the course discussions could be improved not only by all faculty sharing basic background information on the research topic, but by also ensuring faculty know the material taught in the discussion workshop. We would also not know about suggestions for integrating the workshop with the faculty‐led discussions more closely, such as the idea of having one or more student discussion leaders present during the course's faculty‐run sessions, and the students' preference for using research papers in the discussion workshop. These suggestions can further strengthen student involvement and ownership of the course and the graduate program. Additionally, responsiveness of the program leadership to student‐expressed needs builds trust between students and educators, contributing to a more inclusive learning environment. Currently, IGPPEB leadership and discussion workshop leaders are working to incorporate student suggestions into the course for Spring 2023.

### 4.5 Limitations of the study and future improvements

While recent research has shown that skill sets can be developed within a semester‐long initiative,^32^ we acknowledge that this study only examines a small cohort across two academic years. The anonymous survey design did not allow for examining individual differences from pre‐ to post‐course to long‐term surveys. We will improve the survey design to allow individual progress tracking over time. These improvements will allow us to determine whether the baseline experiences of students and pre‐course comfort levels with inclusive discussions are correlated with post‐course changes across variables. Future evaluations can identify specific ways for student leaders to build their teaching and discussion facilitation skills, which will promote these skills during training in the IGPPEB. Continuing to collect evaluation data will allow us to better understand how this course's content and discussion skills development can influence our trainees longitudinally.

Although all students reported in the post‐course survey that the workshop was helpful and should be offered to future cohorts, individual students had different comfort levels contributing to discussions, both before and after the workshop. To increase the perceived improvement in the comfort levels of students in a variety of areas (Figure 2), we will better integrate the skills presented during the discussion workshop with the faculty‐led discussion sessions. We will achieve this, in part, by ensuring that all faculty are aware of the goals and content of the discussion course so that they can better foster the growth of those skills. Differences in the discussion abilities of cohorts fluctuate from year to year and understanding these fluctuations in skills will allow student leaders of the workshop to focus on developing the areas with the greatest reported need. In addition, in response to feedback, the IGPPEB leadership is working with discussion leaders to implement suggested improvements. For example, the IGPPEB leadership is planning to give more ownership over the discussion workshop and subsequent course discussion sessions to student leaders in the future.

## 5 CONCLUSION

The discussion workshop series was created because IGPPEB graduate students identified an important gap in the skill sets required for students to succeed in the program's required discussion course on interdisciplinary scientific topics. The students proposed to start the class with a student‐led discussion workshop to improve students' discussion skills, which also provides student leaders with excellent leadership and professional development opportunities. The discussion workshop resulted in perceived improvement in the four skills areas and agreement among participants that it should continue to be offered. The workshop also proved valuable for building a cohesive cohort within the interdisciplinary graduate program by enabling students to become well‐acquainted with each other. Iterative evaluation is helpful in identifying ways to not only improve students' experiences in the course, but also further strengthen the *espirit de corps* within the IGPPEB community. The increased cohesion in the IGPPEB could lead to additional interdisciplinary research collaborations, enhanced participation in science outreach efforts, and the development of additional student‐driven initiatives.

## AUTHOR CONTRIBUTIONS

C. Shipps designed and led the discussion workshop, revamped the discussion workshop, designed the workshop assessment plan, collected data, and wrote and edited the manuscript; K. L. Thrush revamped and led the discussion workshop, designed the workshop assessment plan, collected data, prepared the supporting information, and edited the manuscript; C. R. Reinhardt designed and led the discussion workshop, designed the workshop assessment plan, and edited the manuscript; S. A. Siwiecki revamped the discussion workshop, prepared the supporting information, and edited the manuscript; J. L. Claydon analyzed the data, wrote and edited the manuscript; D. B. Noble: analyzed the data and wrote and edited the manuscript, and prepared the supporting information; and C. S. O'Hern edited the manuscript.

## CONFLICTS OF INTEREST

The authors have stated explicitly that there are no conflicts of interest in connection with this article.

## Supporting information


Appendix S1

Appendix S2
