# Supplementary material for: “Student‐led workshop strengthens perceived discussion skills and community in an interdisciplinary graduate program”
Source: FASEB Bioadv. 2022 Oct 28;5(1):1–12. doi: 10.1096/fba.2021-00165 (PMC9832528; doi:10.1096/fba.2021-00165)
Supplement: Supplementary file 1 — Appendix S1 [file FBA2-5-1-s002.pdf]

Supporting Information

This word document includes the following supporting information:

Pages 2-4 “Pre-Course Discussion Workshop Survey”: Participants in the IGPPEB course Methods and Logic in Interdisciplinary Research took this pre-course survey before the start of the discussion workshop and course.

Pages 5-7 “Post-Course Discussion Workshop Survey”: Participants in the IGPPEB course Methods and Logic in Interdisciplinary Research took this post-course survey that after the completion of the course.

Pages 8-11 “Long-Term Survey”: Participants in the IGPPEB course Methods and Logic in Interdisciplinary Research took this long-term survey more than one year after the completion of the course.

Pages 12-14 “IGPPEB Methods and Logic in Interdisciplinary Research Course Syllabus”: This is the 2022 course syllabus for the IGPPEB course Methods and Logic in Interdisciplinary Research.

Pages 15-16 “Material covered in each session of the discussion workshop”: This is a detailed description of what was covered in each of the four sessions of the discussion workshop.

Page 17 “References”: References for Supporting Information

Pre-Course Discussion Workshop Survey

Student-Led Workshop in Effective Communication

One of the academic requirements for incoming IGPPEB students is a discussion-based class, Methods and Logic in Interdisciplinary Research, which is aimed at building skills to participate in respectful, cross-disciplinary academic discussions. In the course, students are expected to read and discuss research papers from varying fields every week in peer and faculty groups. Peer-based discussions can often be inefficient due to conversation dominance and underdeveloped skills in referencing the text. Students agree that these qualities can undermine the benefits of peer sessions. To address the diverse intellectual backgrounds and experiences of our incoming graduate students, a senior-student-led workshop has been offered as an additional element in the Methods and Logic in Interdisciplinary Research course. Older students will lead a four-session workshop which will focus on improving:

i) Visual engagement and acknowledgment of peers
ii) Text and evidence-based claims
iii) Redirecting and entering conversation constructively
iv) Peer encouragement

These aims will be addressed through skill-building exercises and class discussion of articles focused on science-related issues that all students will have exposure to at some time in their career, such as reproducibility, career placement, funding, and diversity. Leaders of the sessions will draw from their own course experiences to train newer students on effective communication. The addition of this workshop to the course will strengthen each student's discussion skills that are necessary for a successful graduate and professional career.

---

* Required

Questions

1. What experiences in academic discussions have you had before and how comfortable are you in these settings? Discussion-based classes and relaxed seminars are some examples of academic discussions. *

2. Choose the size of your undergraduate institution: *

Mark only one oval.

- Less than 1000 students
- Between 1000 and 10,000 students
- More than 10,000 students
- Other:

3. How many discussion-centered courses have you had in the past? Discussion-centered means that the class consists of mainly students leading an ongoing discussion focused on a text or other topic, with little direct intervention from a professor. Enter a number. *

4. Were most of your classes small, discussion-centered courses? *

Mark only one oval.

- Yes
- No
- In Between
- N/A

5. Have you had much experience in reading research papers? *

Mark only one oval.

- Yes
- No
- In Between
- N/A

6. Group Speaking: 5=Very, 1= Not Very *

Mark only one oval per row.

	5	4	3	2	1
How comfortable are you speaking in class?	⬭	⬭	⬭	⬭	⬭
How well do you feel you perform in group projects?	⬭	⬭	⬭	⬭	⬭
How well do you feel others perform in your group projects?	⬭	⬭	⬭	⬭	⬭
How comfortable are you in establishing eye contact in group settings?	⬭	⬭	⬭	⬭	⬭
How comfortable are you with initiating a discussion on a new topic?	⬭	⬭	⬭	⬭	⬭
How comfortable are you in asking for help?	⬭	⬭	⬭	⬭	⬭

7. What is your initial perception of this workshop? *

Mark only one oval.

- ⬭ It will be helpful to me
- ⬭ I'm not sure if it will be helpful
- ⬭ It will not help me
- ⬭ It seems like a good idea but it won't help me personally
- ⬭ It doesn't seem like a good idea and won't be helpful at all

Post-Course Discussion Workshop Survey

As a part of the Methods and Logic in Interdisciplinary Research course required for IGPPEB students, you participated in a brief workshop that aimed to develop communication skills important for participating in academic discussions. The workshop met four times. Each time we started with a game or activity that worked on a specific skill, then engaged in discussion about an article chosen by the workshop leaders. We focused on the following four skills:

1) Visual Contact: Who's Afraid of Peer Review
2) Citing the Text: CRISPR Baby Articles
3) Constructive Conversation Entrances: Sexual Harassment Letters Published in Science
4) Peer Encouragement: Justin Chen Ph.D. student Perspective

---

* Required

Questions

1. With regard to your experience in the Methods and Logic in Interdisciplinary Research course please evaluate the following using the following scale: 5=Very Comfortable, 4=Comfortable, 3=Somewhat Comfortable, 2=Not Very Comfortable, 1=Not At All. *

Mark only one oval per row.

	5	4	3	2	1
How comfortable were you speaking in class?	⬭	⬭	⬭	⬭	⬭
How comfortable were you in establishing eye contact in class?	⬭	⬭	⬭	⬭	⬭
How comfortable were you with initiating a discussion on a new topic?	⬭	⬭	⬭	⬭	⬭
How comfortable were you in asking for help?	⬭	⬭	⬭	⬭	⬭

2. What is your perception of the discussion workshop now that you have finished the Methods and Logic in Interdisciplinary Research course? * *Mark only one oval.*

- It was helpful to me
- I'm not sure if it was helpful
- It did not help me
- It seems like a good idea but didn't help me personally
- It didn't seem like a good idea and wasn't helpful at all

3. Why was the workshop helpful or not helpful? Please explain. *

4. Did taking the workshop help you in any other classes or settings (other than Methods and Logic in Interdisciplinary Research)? * *Mark only one oval.*

- Yes
- No

5. If you answered "Yes" above, please describe how the workshop helped you in other classes/settings. If you answered "No" type N/A. *

6. Do you think the Discussion Workshop should be offered again? *

Mark only one oval.

- Yes
- No
- Yes, but with modifications (elaborate below)

7. If the workshop were offered again in the future, what changes would you suggest to improve it? *

Long-Term Survey

The following questions concern the Discussion Workshop you attended as part of the Methods and Logic in Interdisciplinary Research Course that is required for the IGPPEB. For the questions that require a numerical score, you can move the slider continuously between scores of 0 and 100.

The Discussion Workshop has impacted my graduate student experience in the following way:

Extremely negative	Somewhat negative	Neither positive nor negative	Somewhat positive	Extremely positive

0 10 20 30 40 50 60 70 80 90 100

Workshop skills

During the discussion workshop, we practiced four major skills. Which do you feel that you actively use now? (You may select multiple answers.)

- ☐ Visual Engagement
- ☐ Constructive Conversation Entrances
- ☐ Citing the Text
- ☐ Encouragement of Peers
- ☐ None

Regarding the use of <u>visual engagement</u>, how often do you use this skill?

Never	Once a week	2-3 times a week	4-6 times a week	Daily

0 10 20 30 40 50 60 70 80 90 100

I use this skill...

The IGPPEB discussion workshop helped me improve this skill.

Strongly disagree		Somewhat disagree		Neither agree nor disagree		Somewhat agree		Strongly agree		
0	10	20	30	40	50	60	70	80	90	100

Where would you say that you implement this skill in your life as a graduate student?

Regarding the use of <u>constructive conversation entrances</u>, how often do you use this skill?

	Never		Once a week		2-3 times a week		4-6 times a week			Daily
	0	10	20	30	40	50	60	70	80	90	100
I use this skill...											

The IGPPEB discussion workshop helped me improve this skill.

Strongly disagree		Somewhat disagree		Neither agree nor disagree		Somewhat agree		Strongly agree		
0	10	20	30	40	50	60	70	80	90	100

Where would you say that you implement this skill in your life as a graduate student?

Regarding the use of <u>citing the text</u>, how often do you use this skill?

	Never		Once a week		2-3 times a week		4-6 times a week			Daily
	0	10	20	30	40	50	60	70	80	90	100
I use this skill...											

The IGPPEB discussion workshop helped me improve this skill

Strongly disagree		Somewhat disagree		Neither agree nor disagree		Somewhat agree		Strongly agree		
0	10	20	30	40	50	60	70	80	90	100

Where would you say that you implement this skill in your life as a graduate student?

Regarding the use of encouraging your peers, how often do you use this skill?

	Never		Once a week		2-3 times a week		4-6 times a week			Daily
	0	10	20	30	40	50	60	70	80	90	100

I use this skill...

The IGPPEB discussion workshop helped me improve this skill

Strongly disagree		Somewhat disagree		Neither agree nor disagree		Somewhat agree		Strongly agree	
0	1	2	3	4	5	6	7	8	9	10

Where would you say that you implement this skill in your life as a graduate student?

Workshop Evaluation

	Strongly disagree		Somewhat disagree		Neither agree nor disagree		Somewhat agree		Strongly agree		
	0	10	20	30	40	50	60	70	80	90	100
The workshop helped me feel more prepared for scientific discussions.											
The workshop helped me have more constructive conversations.											
The workshop helped me feel more welcomed in the IGPPEB community as a whole.											
The workshop helped me feel more confident in discussions.											

If you could change the workshop, what would you change?

Is there anything else you would like us to know?

<u>Course Instructors:</u>
For a complete list, please see the Canvas course website.
Overall: Corey O'Hern; Dorottya Noble
Administrative Assistant: Peggy Eatherton
Discussion Workshop Leaders: Kyra Thrush; Sara Siwiecki

<u>Course Overview:</u> The class meets **5:30 to 7:30 pm** (or until discussions are complete) on Monday and Wednesday in **405 Bass Laboratory**. The start time is chosen to allow students and faculty to attend 4pm research seminars without a conflict. **This is a required course for IGPPEB students.**

The papers to be covered will be posted on Canvas – in the Files section with a folder for each session.

Typically, students cover one pair of papers per week, and meet to analyze and discuss them **without the Professors during the Monday session**. At the Monday session, students should combine their varied expertise to make sure that everyone understands all aspects of the papers. Participation in the Monday sessions is an integral component of the course and is mandatory. All students should sign-in on Monday to verify attendance with the week's discussion leaders, who will send a digital copy of the attendance sheet to the administrative assistant. Students further discuss the papers **with the Professors in the Wednesday sessions**.

The class will start off with an IGPPEB student-led Communications Workshop, aimed at increasing the discussion skills of students participating in the course.

<u>Learning Objectives:</u> Following successful completion of this course, *including a Discussion and Communication Workshop, student-led paper discussions, professor-led paper discussion, and writing and pitching a follow-up experiment*, students will be able to

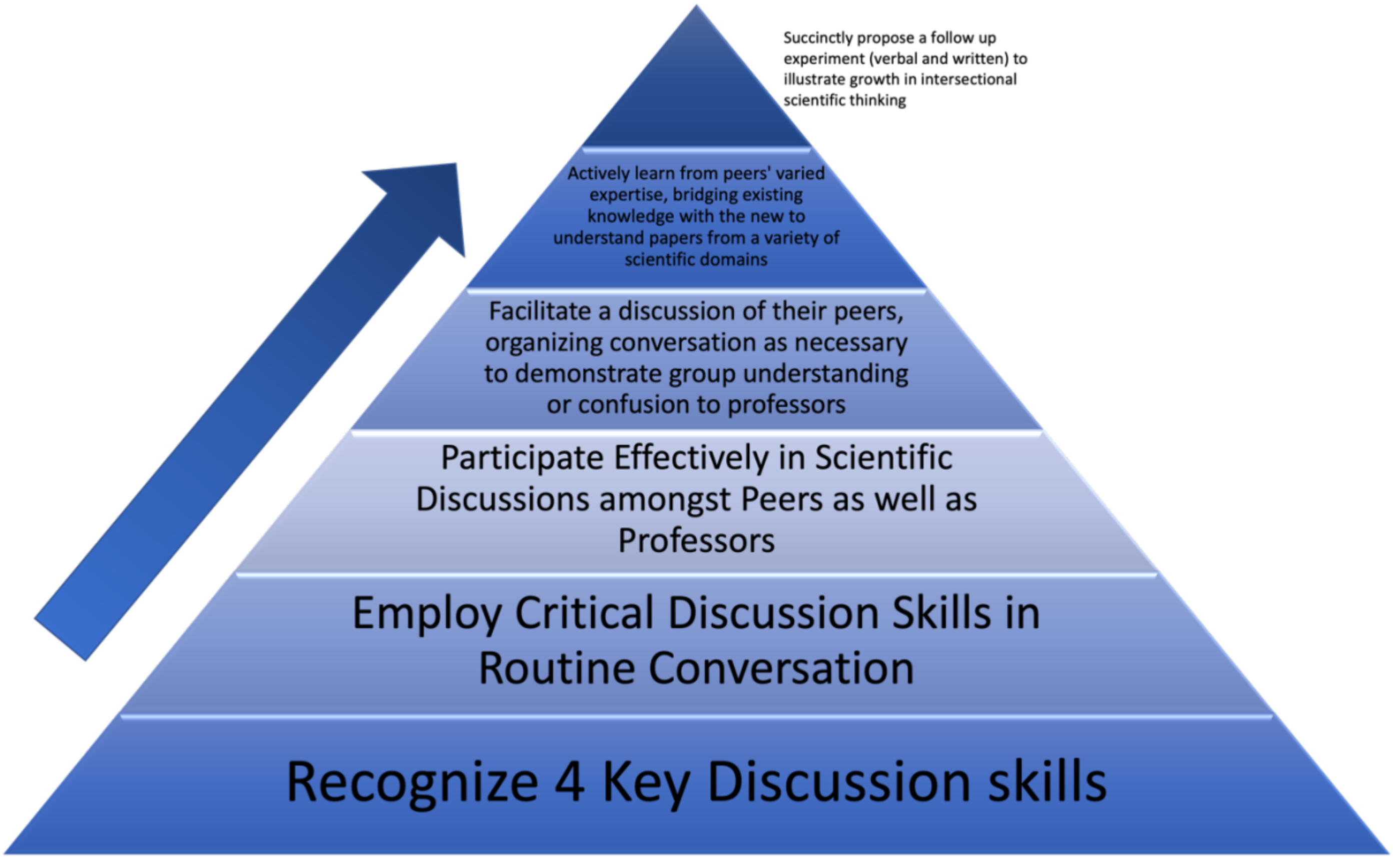

Grades: Reflecting the above learning goals, grades for the course will be broken down as follows:

Participation in Discussion Workshop sessions, student-led, and professor-led sessions<br>• Demonstrating the 4 discussion skills presented during the workshop<br>• Contributing to the discussion according to your own learning & experience<br>• Listening to others' expertise and incorporating these thoughts<br>• Working with others to answer any questions proposed by peers or the professors<br>• When necessary, submit additional unanswered questions to Canvas	60%
Acting as discussion facilitator during assigned week<br>• Take and submit attendance to the administrative assistant<br>• Gather outstanding questions from students following Mondays' Peer Discussion<br>• Submit outstanding questions to Canvas for the week<br>• Remind professors about the outstanding questions for the week<br>• Where appropriate, remind other students of the need to use discussion skills for improved discussion	10%
Final presentation and abstract submission<br>• **Final presentations, with faculty present, will be held April 4-13th. Each student needs to only attend their assigned session.**<br>• Students are *strongly encouraged* to practice with each other prior to their final presentations.<br>• More details regarding the abstracts are found below	30%

Abstract and Presentation: Students will choose any paper that is covered in class and will devise an additional experiment, simulation, or calculation as a follow-up.

Abstract: Students should write a ***finely polished abstract - including a title.*** More details can be found in the Abstract & Presentation folder on Canvas.
Presentation: The presentation will be 15 minutes per student, including questions. Please anticipate numerous questions, so plan to speak for approximately **10-12** minutes only). We will be strict with the timing - because the ability to communicate your message in a small number of words, or a short amount of time, is an important skill. Students can use PowerPoint, the blackboard, or any presentation style they desire. In the presentation, students should explain 1) the experiment they propose, 2) why it is important (cover enough background of the paper to understand the significance of the proposed additional study), 3) how they would perform it, and 4) how they would interpret the results.

Schedule for Methods and Logic in Interdisciplinary Research, including the initial Communications Workshop led by IGPPEB students Kyra Thrush and Sara Siwiecki.

,

Jan 24	Introduction, course overview, and session 1 of the Communications Workshop: **Visual Engagement**
Jan 26	Session 2 of Communications Workshop: **Citing Text**
Jan 31	Session 3 of Communications Workshop: **Constructive Conversation Entrances**
Feb 2	Session 4 of Communications Workshop: **Encouragement of Peers**
Feb 7-9	Professor Pair 1
Feb 14-16	Professor Pair 2
Feb 21-23	Professor Pair 3

Feb 28- Mar 2	Professor Pair 4
Mar 7-9	Professor Pair 5
Mar 14-16	Professor Pair 6
**Apr 4-13**	**Student Presentations**
**Apr 25**	**Abstracts due – please submit on Canvas**

“Material covered in each session of the discussion workshop”

The material covered in each of the four sessions of the discussion workshop is described below:

1) *Visual engagement:* The learning objectives of this session are 1) to address peers by name, 2) to improve eye contact while speaking and avoid speaking only to the discussion leaders, and 3) to gauge facial cues. The engagement strategy for objectives 1 and 2 is a ‘name game’, in which students first state their name and a personal fact (i.e. color of the socks they are wearing) and then repeat the names and facts about all of the students before them until each student has participated. The engagement strategy for objectives 2 and 3 is often referred to as ‘zen counting’, an exercise in which students compare the ease with which, as a group, they can count to a specified number (N ≥ number of students) under two different conditions: in one condition, students turn their backs to each other so they cannot see each other’s faces, and in the other condition, they face forward, allowing the use of visual cues. All students must speak, and the challenge is that students must start over if two students speak at once, emphasizing the importance of body language cues for efficient gameplay. It is well-known that this activity encourages interactions in discussion-centered courses[1]. Following these games, the students discuss an article about scientific peer review[2], addressing each other by name and following conversation visual cues, thus practicing the learning objectives of the session.

2) *Citing the text:* The learning objective of this session is to refer to relevant parts of the text during a discussion so that others can easily follow. After playing the ‘name game’ once again, as a warm-up exercise, students play the ‘cracking the code’ game. In this game, small teams of students, all with a common base text such as *Little Red Riding Hood[3]*, create an alphanumeric code to convey a short message using words from the text to another student team. This code is not discussed or standardized among the larger group prior to gameplay. The next team must interpret the alphanumeric code, then recode their interpretation into a drawing to pass on to the final team. The final team then must crack the code to find the original short message. Student teams compare the ease of translating a code consisting of only symbols or pictures to a code that permits referencing lines of text. The students then discuss how the use of ambiguous codes can lead to confusion in understanding, underscoring the need for consistent and clear citations during discussions. This exercise is immediately followed by a discussion of an academic article, focusing on scientific ethics, where students practice clear citations of the text[4, 5].

3) *Constructive conversation entrances:* The learning objectives of this session are 1) to contribute to a discussion by logically following up what others have said, and 2) to think of a class discussion as a group endeavor that builds on collective ideas and understanding. Students play the ‘evolving story’ game, in which students are pre-assigned words that they must use in a sentence to contribute to an ongoing group story. While students can assist others with filler sentences, the game can only conclude when all words have been used and a meaningful story is constructed. The game is played multiple times and students compare the story quality and game difficulty when the words are unified by a common theme versus randomized. Afterwards, students discuss a controversial scientific editorial where they practice constructive conversation entrances and engage with others’ points rather than simply interjecting their own[6, 7].

4) *Encouragement of peers:* The learning objectives of this session are 1) to recognize when peers may need encouragement to participate in a discussion and 2) to help them participate. Students play the ‘secret signal’ game, which is a twist on the classic game of ‘spoons’. All students are paired up and sit across the table from their partner. Each student starts with 4 playing cards. Using 2 or more decks, cards are passed around the table one-by-one, allowing a student to exchange a card if so desired, with the objective of getting 4 of a kind. Once this is achieved, the student must give the predetermined secret signal to their partner, who must announce their partner has 4 of a kind. This constitutes a point for the pair. If, however, someone else in the group thinks they saw a signal for a winning hand, they can interject before the partner’s announcement, thus negating the win, and scoring 2 points for the interjecting pair. If they are wrong and they falsely accuse a pair of winning, they lose 1 point. Students learn to observe body language of the entire room while having both a personal and team agenda.

Having gained awareness of other students in the room, students then discuss a paper on a controversial topic[8] and pay special attention to peers who want to contribute and then encourage them to do so.

“References”

1. Smith, L.A. and M. Foley, *Partners in a Human Enterprise: Harkness Teaching in the History Classroom.* The History Teacher, 2009. **42**(4): p. 477-496.
2. Bohannon, J., *Who's afraid of peer review?* Science, 2013. **342**(6154): p. 60-5.
3. Grimm, B. *Little Red Riding Hood*. [cited 2019.
4. *How to respond to CRISPR babies.* Nature, 2018. **564**(7734): p. 5-5.
5. Belluz, J., *Is the CRISPR baby controversy the start of a terrifying new chapter in gene editing?*, in *Vox*. 2018.
6. Li, J.R., S. Walker, J.B. Nie, and X.Q. Zhang, *Experiments that led to the first gene-edited babies: the ethical failings and the urgent need for better governance.* Journal of Zhejiang University-Science B, 2019. **20**(1): p. 32-38.
7. Starr, D., *Fighting words.* Science, 2020. **367**(6473): p. 16-19.
8. Chen, J., *Coming to terms with six years in science: obsession, isolation, and moments of wonder*. 2018: Stat news.
